# Use of Lumason Contrast Echocardiography in Post-myocardial Infarction Ventricular Septal Defect

**DOI:** 10.7759/cureus.27128

**Published:** 2022-07-21

**Authors:** Michael Cinelli, Boutros Karam, Jonathan Spagnola, Marc Assaad, Chadi Salmane, Wissam Hoyek, Charles Schwartz

**Affiliations:** 1 Cardiology, Staten Island University Hospital, Staten Island, USA; 2 Internal Medicine, Staten Island University Hospital, Staten Island, USA; 3 Interventional Cardiology, Northwell Health, New York, USA

**Keywords:** transthoracic echocardiography, cardiac surgery, lumason, acute myocardial infarction, contrast echocardiography, ventricular septal defect

## Abstract

We report herein the case of an elderly female who presented with myocardial infarction complicated by ventricular septal defect (VSD) that was evident on cardiac auscultation and contrast echocardiography using Lumason® (Bracco Diagnostics Inc, Monroe Township, USA). Patient underwent surgical repair for her VSD post-infarct along with coronary artery bypass grafting after being treated for cardiogenic shock. We also highlight the management strategies in patients with similar complications. In this report, we shed the light on the importance of using Lumason contrast for the identification of shunt and for the diagnosis of VSD. Lumason contrast is widely available and licensed.

## Introduction

Ventricular septal defect (VSD) is a known complication seen after acute myocardial infarction (MI), particularly anterior MI or lesions of the left anterior descending artery (LAD), and inferior MI or lesions of the right coronary artery (RCA) [1]. VSD occurs in an almost equal percentage in RCA and LAD lesions according to the SHOCK trial [1]. Identification of a VSD post-MI is classically diagnosed on physical exam as a holosystolic murmur along the left lower sternal border with a palpable thrill. We present a case of a female patient presenting with acute anterior wall MI found to have a murmur characteristic of VSD identified on auscultation, which was only demonstrated on echocardiography with the addition of Lumason® (Bracco Diagnostics Inc, Monroe Township, USA) contrast, which ultimately required surgical repair.

## Case presentation

**Figure 1:**
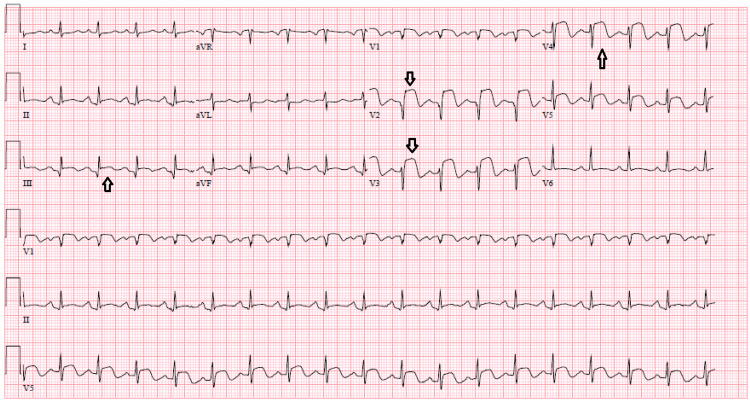
ECG showing ST segment elevations in the anterior and inferior leads.

A 76-year-old female with a past medical history of coronary artery disease status post percutaneous intervention to the LAD, hypertension, and dyslipidemia presented to the emergency department with continuous, knife-like pleuritic chest pain that started a few days prior to presentation. It was associated with intermittent backache and headache, worse with deep breathing and coughing. Blood pressure (BP) on arrival was 223/115 mmHg and electrocardiogram (ECG) showed diffuse ST elevations. Computed tomography angiographic (CTA) scan of the chest ruled out aortic dissection. Transthoracic echocardiography (TTE) showed a left ventricular ejection fraction (LVEF) of 40-45%, multiple left ventricular regional wall motion abnormalities (anterolateral, apico-inferior, and apical), and grade I diastolic dysfunction. The initial troponin was 0.16 and the repeat was 1.14. Repeat ECG showed ST elevation in anterior and inferior leads (Figure 1). She was loaded with clopidogrel and taken for emergent cardiac catheterization, which showed 100% occlusion in the mid-LAD, 99% in the 1st obtuse marginal artery, and 80% in the right posterior descending artery. Two drug-eluting stents were placed in the mid-LAD.

**Figure 2:**
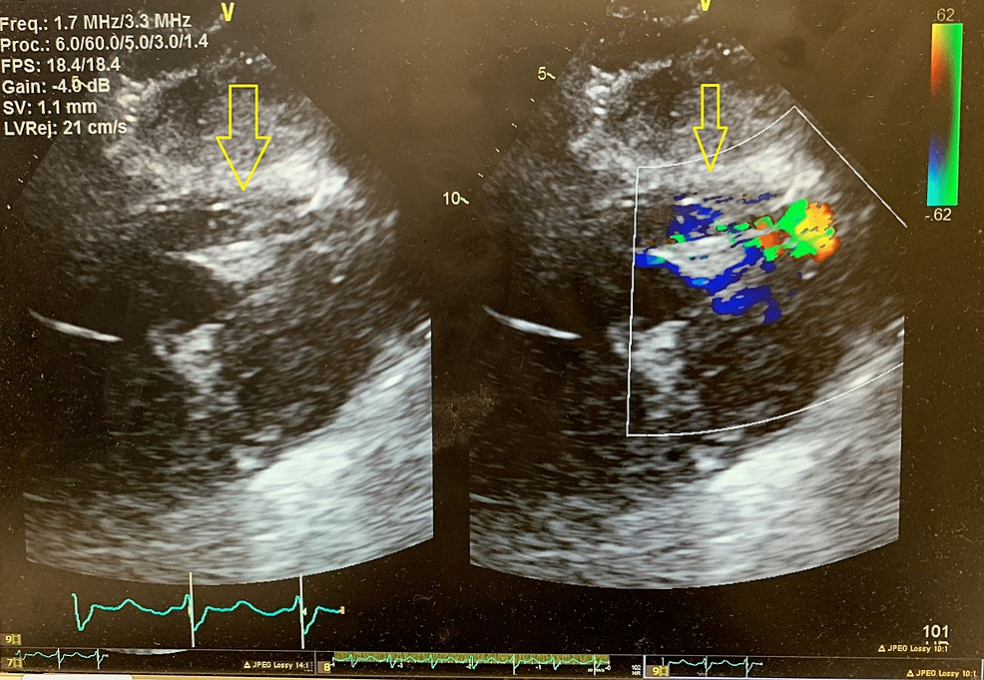
Color Doppler on transthoracic echocardiogram showing a moderately sized anteroapical ventricular septal defect with bidirectional shunting, predominantly left to right.

**Figure 3:**
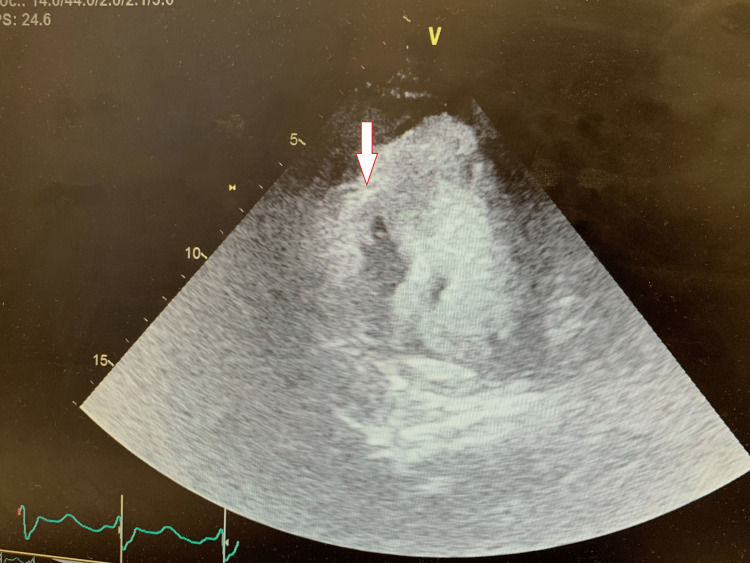
Transthoracic contrast echocardiography showing the Lumason crossing through the ventricular septal defect with a left to right shunt.

**Figure 4:**
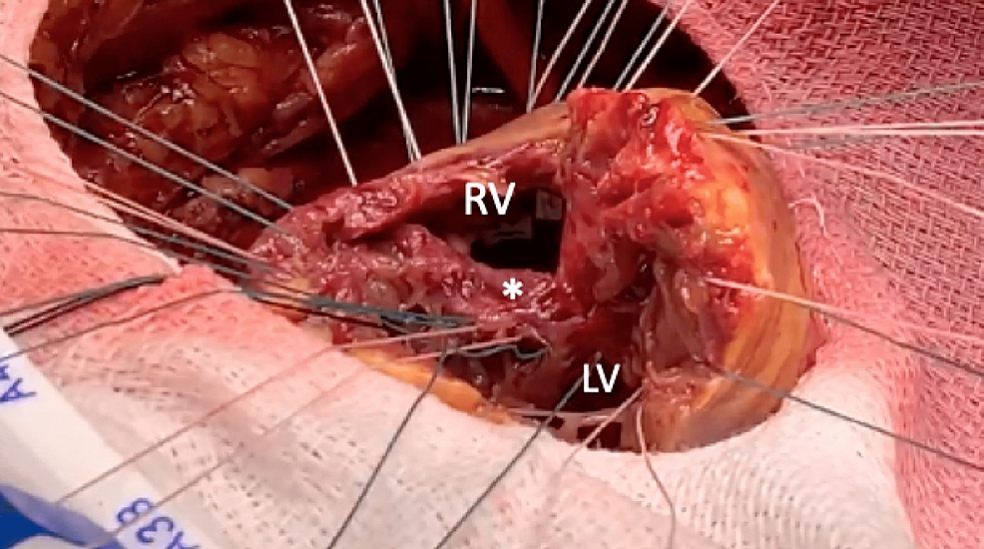
Intra-operative exposure of ventricular septal defect (white asterisk) between the right ventricle and left ventricle.

**Figure 5:**
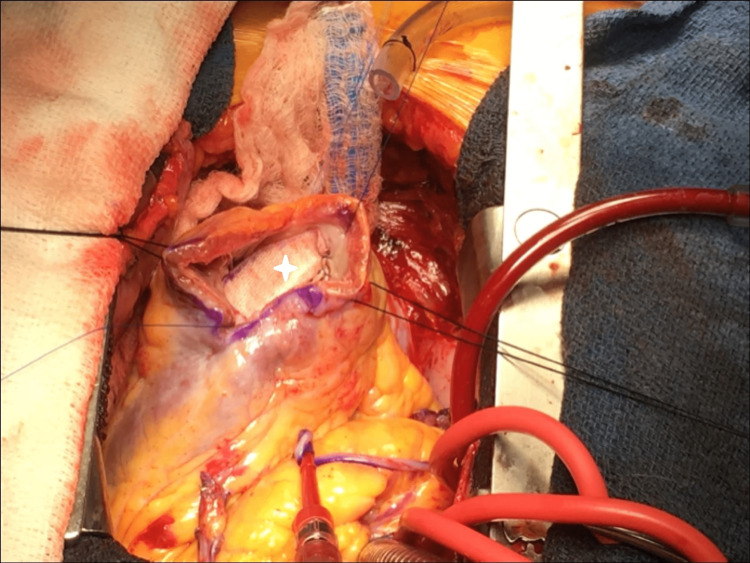
Intra-operative ventricular septal defect after surgical repair with a patch (white asterisk).

On the same night, the patient became hypotensive with a mean arterial pressure of <50 mmHg, a heart rate of 62 beat per minute, and BP of 64/40 mmHg, consistent with cardiogenic shock. Phenylephrine was initiated. Physical exam was notable for a loud systolic murmur over the left lower sternal border. Bedside TTE showed a hyperdynamic left ventricle and apical hypokinesis. There was no mitral regurgitation. Color Doppler across the septum failed to reveal any shunt. Given the evidence of a loud holosystolic murmur on exam and the rapid clinical deterioration, there was a high index of suspicion for a ventricular septal defect (VSD). A repeat TTE was done with Lumason contrast for further assessment of mechanical complications. It showed a moderately sized anteroapical VSD with bidirectional shunting, predominantly left to right (Figures 2-3). Transesophageal echocardiography (TEE) was not performed due to the patient's instability, and the diagnosis of VSD was made using the Lumason contrast. The peak velocity of the left to right shunt was 3.92 m/s (61 mmHg peak gradient) and the right to left shunt was 2.3 m/s (21 mmHg peak gradient). The patient was brought back to the cardiac catheterization lab for intra-aortic balloon pump (IABP) placement and was subsequently started on venoarterial extracorporeal membrane oxygenation (VA-ECMO) with left femoral vein and right subclavian artery access due to a refractory shock state. Surgical repair of the VSD was delayed due to instability, thereby allowing for recovery of the myocardium optimal for surgical repair. Six days later, the patient underwent surgical VSD repair with a patch (Figures 4-5) and coronary artery bypass grafting. She was removed from VA-ECMO with improvement.

## Discussion

VSD is a well-known complication of MI in the first two weeks following the event. The degree of the VSD size varies and is evidenced by the murmur intensity. Imaging modalities to identify this complication require a high degree of clinical suspicion from physical exam. Transthoracic echocardiography (TTE) is pivotal in the diagnosis of VSD, particularly with the use of Doppler as it allows for the calculation of the size as well as the hemodynamic parameters of the VSD [2]. Contrast echocardiography, while previously used more widely for VSD detection [3], is rarely used today since the advent of color Doppler [4]. It is however used for the detection of residual shunting after VSD repair [4]. Our patient’s VSD was not clearly evident on the initial TTE and was definitively revealed with the use of Lumason contrast echocardiography.

Lumason contrast has been widely used in echocardiography for the detection of intracardiac thrombus and shunts [5]. The use of contrast was previously contraindicated in intracardiac shunts due to concerns about the risk of cerebral ischemia due to microvascular obstruction during the injection of the contrast [6,7]. However there has been strong evidence that it is safe for use in shunts as the particle size of the contrast media is comparable to that of agitated saline, which is routinely used in the evaluation of intracardiac shunts [5,8]. We aim to show the usage of Lumason contrast echocardiography in both the diagnosis of VSD and in guiding the approach to repair of the VSD. We also aim to increase awareness and utilization of microbubble contrast echocardiography in both the diagnosis of post-MI VSD as well as to aid in the plan for repair whether surgical or percutaneous by better anatomy identification. It can both make practitioners aware of complex anatomy and can help make the decision between the two repair entities.

Transesophageal echocardiography is indicated in inferior MI with VSD where percutaneous repair is considered [2]. Surgical repair and closure is the gold standard treatment for post-MI VSD [2]. The timing of surgical repair is highly debatable depending on hemodynamic status. Patients who are stable hemodynamically are often repaired early, however, in patients with complex anatomy, repair may be delayed due to tissue compromise from the MI [2]. Delayed repair (5-7 days post-MI) has shown a mortality benefit as there is time for adequate tissue repair and reorganization and as such is currently the guideline recommended for patients with post-MI VSD [2,9]. Our patient required surgical repair, and the prompt identification of the VSD facilitated appropriate management and survival of the patient. A multidisciplinary team of cardiologists, interventional cardiologists, heart cardiologists, and cardiothoracic surgeons should help formulate an individualized repair management strategy. Contrast echocardiography with Lumason can guide this team and guide the approach of repair in this precarious patient population by identifying the site and the position of the VSD.

## Conclusions

VSD is a jarring complication of MI. Multiple imaging modalities have been used to identify and guide the management of this potentially fatal sequela of acute MI. We demonstrate the use of contrast echocardiography with Lumason in the detection of a VSD post-MI in a patient whose TTE did not clearly show the defect. We further emphasize the use of microbubble contrast echocardiography serving as both a diagnostic aid as well as a key tool to guide the approach of percutaneous or open surgery to repair post-MI VSD. Management of this pathology requires multimodal imaging as well as collaboration among interventional cardiologists, structural cardiologists, and cardiothoracic surgeons. More studies should be done to help support the result of this report.
